# Utilizing community engagement studios to inform clinical trial design at a Center of Excellence for Alzheimer’s Disease

**DOI:** 10.1017/cts.2022.388

**Published:** 2022-04-11

**Authors:** Miriam R. Stock, Mirnova E. Ceïde, David W. Lounsbury, Jessica Zwerling

**Affiliations:** 1 Medical Program, Albert Einstein College of Medicine, Bronx, NY, USA; 2 Department of Psychiatry & Behavioral Sciences, Albert Einstein College of Medicine, Montefiore Health System, Bronx, NY, USA; 3 Department of Medicine, Montefiore Health System, Bronx, NY, USA; 4 Department of Epidemiology & Population Health, Albert Einstein College of Medicine, Montefiore Health System, Bronx, NY, USA; 5 Department of Neurology, Albert Einstein College of Medicine, Montefiore Health System, Bronx, NY, USA

**Keywords:** stakeholder studio, community engagement, Alzheimer’s disease, virtual intervention

## Abstract

Despite the disproportionate burden of Alzheimer’s disease in older adults of color, the scientific community continues to grapple with underrepresentation of racial and ethnic minorities in clinical research. Our Center of Excellence for Alzheimer’s Disease (CEAD) collaborated with a local community partner to conduct community engagement (CE) studios to effectively involve our community of diverse older adults in the early planning stages of a clinical trial. Given the COVID-19 pandemic, the in-person studio format was adapted to allow for virtual, real-time participation. Our objective is to describe the process and feasibility of conducting virtual CE studios in an older adult population. Ninety percent of participants were non-Hispanic Black community-dwelling woman aged 60 years and older. The overall background and proposed clinical trial design was presented to the participants who then made recommendations regarding potential recruitment strategies, the use of culturally relevant language to describe the study, and logistical recommendations to improve participation and retention among community members. Our CEAD successfully conducted virtual CE studios during the COVID-19 pandemic, by partnering with a community-based organization, to engage community stakeholders about clinical trial design. CEADs are in a unique position to implement CE studios to better support patient access to clinical trials.

## Introduction

Alzheimer’s disease (AD) affects approximately 6.2 million individuals in the USA [1]. This number is projected to increase to a staggering 13.8 million individuals by 2060 [2]. It has been well established that the prevalence and incidence of AD is higher among non-Hispanic Black or African-American (NH Black) and Hispanic older adults as compared to non-Hispanic Whites (NH White) [3–5]. Despite the disproportionate burden of AD in older adults of color, the scientific community continues to grapple with the underrepresentation of racial and ethnic minorities in clinical research [6–8].

### Overview of the New York State Center of Excellence for Alzheimer’s Disease (CEAD)

The Hudson Valley a Center of Excellence for Alzheimer’s Disease (CEAD) is one of 10 Alzheimer’s Disease Centers of Excellence supported in part by a grant from the New York State Department of Health in an ambitious program that aims  montefiore.org/Alzheimer’s-center [9] to:

1. Expand knowledge about AD and related dementias.
2. Improve access to screening, diagnosis, and clinical trial opportunities for patients.
3. Provide community-based support services for them and their caregivers.
4. Offer training programs for providers in all clinical disciplines.

The CEAD provides outpatient-based multispecialty dementia care utilizing a consultative model, in which patients undergo a comprehensive three-step evaluation by a geriatrician, neurologist, and neuropsychologist with support provided by geriatric psychiatry, physiatry, and social work. The majority of the clinical evaluations for the CEAD are conducted at the Center for the Aging Brain located in Yonkers, New York. Our patient population largely originates from Bronx County and seven counties in the Hudson Valley region: Westchester, Rockland, Putnam, Dutchess, Sullivan, Orange, and Ulster. As previously described, the patient population at the CEAD is diverse with 25% African-American, 18% Hispanic, and 5% multiracial patients [10].

### Clinical Trial Barriers at the CEAD

In line with the CEAD’s goal of increasing patient access to clinical trials, 1,018 of the 1,231 patients (83%) evaluated from January to June 2021 were provided information about clinical trials. Yet only 331 patients (33%) were enrolled in these studies. It is unclear why there is a discrepancy between referral and enrollment in clinical trials, but one component of the problem may lay in the misalignment between the needs of our culturally rich patient population and the demands of established clinical trial design.

This misalignment is apparent in our struggle to study Problem Adaptation Therapy (PATH) at the CEAD. PATH was developed by Kiosses and colleagues at Weill Cornell and is a psychosocial intervention for older adults with depression and cognitive impairment that utilizes environmental adaptations, compensatory strategies, and caregiver involvement [11]. However, PATH was only studied in an English-speaking, non-Hispanic White population with 12 or more years of education [11]. In 2016, the CEAD partnered with Dr. Kiosses to study PATH in our culturally diverse population, running two clinical trials simultaneously. The first study was a PATH open-label patient-centered pilot study. The setting was flexible between home and office, depending on patient preference. PATH was delivered in both English and Spanish based on patient-preferred language [12]. The second study, PATH-Mild Cognitive Impairment (MCI), was a multicenter-randomized control trial (RCT) (1R01AG050515) based out of Weill Cornell in Westchester, NY, Johns Hopkins University in Baltimore, MD, and Montefiore in the Bronx, NY. PATH-MCI was office-based and limited to English-speaking participants. We were able to enroll 145 patients in the PATH pilot study and successfully demonstrated the feasibility of the intervention in our cohort [12]. On the other hand, the Montefiore CEAD site for the PATH RCT struggled to recruit participants to the trial and was ultimately only able to enroll two participants, while Weill Cornell and Johns Hopkins had more successful enrollment.

The stark contrast between our success with the PATH pilot study and our struggle with the PATH RCT reflected several shortcomings. The PATH RCT study did not provide transportation for participants because we did not account for the differing transportation needs of patients from the Bronx as compared to patients from Westchester and Baltimore. In determining inclusion and exclusion criteria for the study, our design did not consider that many Montefiore patients do not speak English as their primary language, nor did they have the same level of education. As such, many of our patients were excluded from participation based on these criteria alone. Moreover, it was likely that there were many additional shortcomings that we were unaware of. We decided to plan community engagement (CE) studios in order to gain insight from stakeholders to inform the design of another trial to study PATH in our community.

### Recognizing Structural Shortcomings in “Non-Collaborative” Research

In 1993, the National Institutes of Health (NIH) Revitalization Act mandated ethnic minority inclusion in randomized clinical trials, defining underrepresented minorities as African-Americans, Latinos, and American Indians, but disparities still persist [13]. Some of the most commonly reported barriers to more diverse research enrollment include mistrust of the medical system, perceived harms, financial cost, transportation concerns, lack of education about the study, fear, time commitment, and the lack of rapport between researchers and the community [14, 15]. In recent years, CE has been recognized as the key to increasing ethnic minority participation and retention in clinical research as well as a means to overcoming many of the aforementioned barriers [13, 16]. This recognition is being propelled by a clear and present understanding of the historical and structural shortcomings of non-collaborative research, whether rooted in epistemological, ideological, and/or pragmatic dilemmas [17].

*Community Engagement (CE) Studios*, initially piloted in 2009 by the Meharry-Vanderbilt Community-Engaged Research Core, are a rapid, low-resource approach to improving research design, implementation, and successful execution [18]. In CE studios, targeted representatives from one or more stakeholder groups (often patients or providers) serve in a consultative role to help researchers address anticipated challenges in study design or implementation [18].

### Conducting CE Studios

Studios have previously been described as in-person, group meetings conducted jointly by a *project lead* and a *neutral facilitator* with 10–12 *community stakeholders* (community experts). The neutral facilitator is responsible for conducting a semi-structured, open discussion between the project lead and participating community stakeholders, to elicit authentic and constructive feedback about the project needs or challenges.

Careful selection and deliberate recruitment of the most appropriate stakeholder groups is essential to the success of a given studio event. *The studio advisor* – ideally a fellow, well-respected faculty member with experience in conducting patient-centered outcomes research, clinical practice improvement processes, community public relations, participatory action research, and/or program evaluation – assists the project lead in choosing and engaging targeted stakeholder groups for participation, ensures that information obtained during a studio event is collected and reported in an ethical and timely manner, and fosters trusting, respective rapport with all participating stakeholders. Recruitment is conducted by one or more *community navigators* who are, ideally, persons with deep knowledge about the targeted community and who are familiar with clinical and translational research designs and methods [18].

Faced with the COVID-19 pandemic, the CEAD developed the Coordinated Care at Risk/Remote Elderly Program [CCARRE] through which it was able to deliver multidisciplinary evaluation and management to patients via a unique telehealth model [19]. Through this pilot program, staff members at the CEAD developed competence in the use of telehealth. With that knowledge in mind, we decided to adapt our previously planned CE studios to a virtual format. In this paper, we describe the process by which we organized and conducted virtual CE studios in order to inform the design of a proposed clinical trial of PATH [11] in a diverse cohort of depressed older adults with cognitive impairment.

## Methods

The Einstein/Montefiore Institute for Clinical and Translational Research (ICTR) has a CE core which seeks to enhance the quality of clinical and translational research through engagement of diverse community and scientific stakeholders. CE studios were scheduled under the advisement of the ICTR faculty, who served as our studio advisor.

The Jewish Association Serving the Aging (JASA) served as our community navigator throughout the project. It is a well-known community organization that has provided critical aging services to older adults in New York City since 1968 (jasa.org). One component of JASA’s services is the Naturally Occurring Retirement Community (NORC) in the Bronx, NY, which offers a wide range of social services, educational and recreational activities, supportive counseling, assistance with securing benefits and entitlements, health-related services, and transportation. In response to the COVID-19 pandemic, JASA NORC converted their ongoing community programming to a virtual format. In particular, their caregiver support and wellness groups continued to meet weekly via Zoom© video conferencing platform (San Jose, CA). They provided devices to their under-resourced clients and IT support for the general NORC population.

**Fig. 1.**
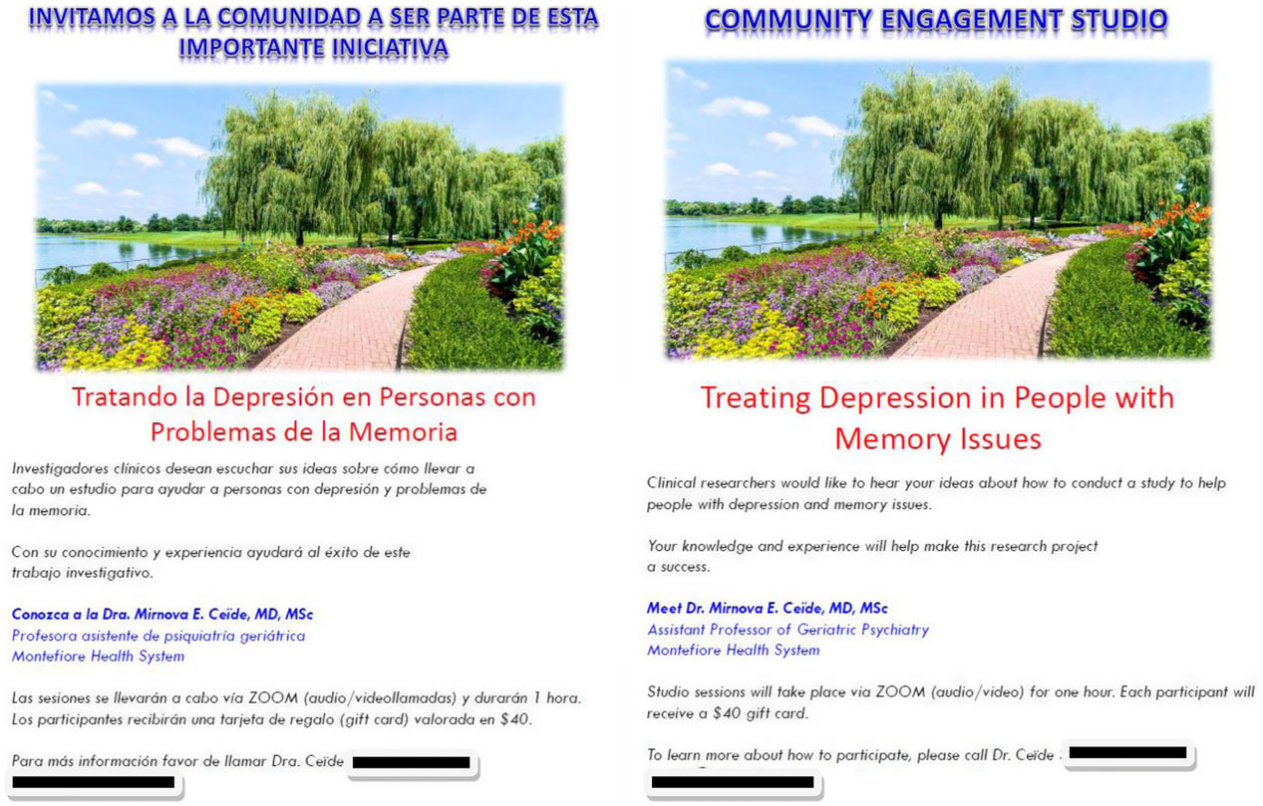
Community engagement studio recruitment flyers.

With the guidance of our studio advisor, the project lead (Dr. Ceïde) contacted the JASA community navigator via introductory emails describing the objectives of the CE studios. The navigator expressed interest and recommended targeted stakeholder groups: the JASA caregiver support group and wellness group. The project lead and the studio advisor developed CE studio flyers (Fig. 1), which were circulated by the navigators.

### Studio Virtual Format

**Fig. 2.**
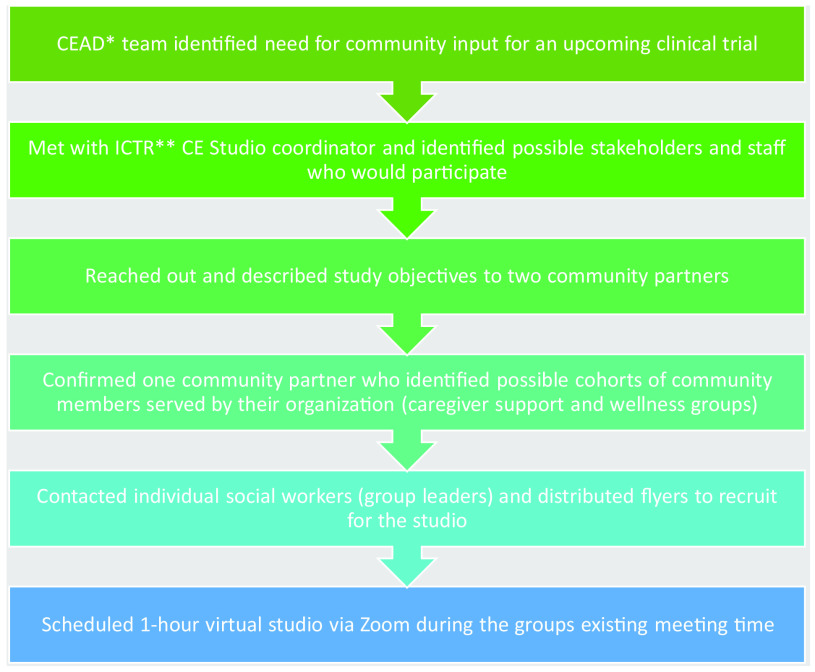
Process of coordinating virtual community engagement (CE) studios. *CEAD: Center of Excellence for Alzheimer’s Disease. **ICTR: Einstein/Montefiore Institute for Clinical and Translational Research.

We conducted the CE studios via Zoom© during the existing JASA NORC virtual caregiver and wellness group meeting times (Fig. 2). IRB approval was waived as the community input was aggregated data.

Each 1-h session began with a brief presentation (7 min) by the project lead. It was presented with minimal medical jargon and at an eighth-grade reading level. The content of the presentation included background on depression in dementia, underrepresentation of diverse populations in clinical research, and the basic aspects of the proposed clinical trial including inclusion/exclusion criteria, limited description of the intervention, and control groups and possible risks. Inclusion criteria included are follows: adults aged 60 years or older, endorsing feeling sad or depressed, having memory complaints impairing daily functioning, having caregiver support, and being fluent in Spanish or English. Exclusion criteria includedare as follows: individual is already in psychotherapy, too medically ill to attend sessions, having suicidal thoughts, or having psychiatric symptoms requiring hospitalization. The PATH intervention was described as structured “talk therapy” and the control was non-problem-focused talk therapy. Both groups would participate in 12 to 15 50-min therapy sessions. We did not indicate what the setting of the therapy sessions would be (i.e. in-person or remote; home or office).

After the initial presentation, the neutral facilitator (the CEAD lead social worker) provided questions to prompt discussion among participants (Table 1). Notes were taken during the session by the project lead. Sessions were recorded for review by the second team member. Participants received compensation for their participation.

**Table 1.** Community engagement studio discussion questions

How is this project important and valuable to older adults in your community?
Who would benefit most from this intervention and how can we reach them?
How do we get people to agree to participate?
How do we get people to stay engaged and complete the program?
Would you need transportation arranged for, or reimbursed?
What kind of compensation is reasonable?
What questions or concerns might you have about engaging in a Talk Therapy program?
How should talk therapy be described?
What would you be looking for in the study counselor?
What questions or concerns would you have about completing memory testing and questionnaires?
What questions or concerns would you have if you were a significant other/caregiver?

## Results

Twenty-one women ≥60 years old who live in the NORC catchment area participated in the CE studios. While the sessions were open to any older adults aged 60 years and older in the NORC, 90% of stakeholder studio participants were non-Hispanic Black women.

### Impact/Significance

Overall, participants felt that the study would be impactful for their community. They noted an increase in depression among frail older adults living in their community, especially during the COVID-19 pandemic. In addition, studio participants who identified as caregivers felt that anything that could make it “easier” to care for their loved one would be helpful to both the participant and the caregiver.

### Recruitment/Engagement

Recruitment could be best achieved by engaging with older adults through public events (e.g. health fairs and flyers in common areas) or advertising in the local pharmacies. Participants noted that partnering with community-based organizations was a good idea. Participant engagement could be promoted through addressing transportation needs, with particular attention paid to distance traveled, and providing refreshments for participants who are coming in for study assessments. They also suggested a local site for study assessments, such as the local senior center.

### Recommendations about the Clinical Trial Design

Participants were open to an in-person, telephone, or video hybrid model. While several participants mentioned concerns that the intervention would be conducted virtually, one participant countered that “even a telephone call can make a difference.” Additionally, the proposed 12 to 15 psychotherapy sessions were felt to be burdensome, while 6 to 8 sessions seemed more feasible to the group. Interestingly, participants had no concerns about completing questionnaires or neuropsychological testing.

When describing the proposed PATH intervention, participants mentioned that there is a stigma with the term “therapy, especially depending on the cultural background.” They recommended using other terms like “helpful conversations” or “chats.”

Participants shared that they would prefer a study therapist who was friendly, open, nonjudgmental, and empathetic. Some participants also suggested a preference for an ethnically concordant clinician, stating “I want them to look like me.”

## Discussion

In this paper, we describe how we utilized CE studios to consult community stakeholders about the design of a proposed clinical trial in its early planning stages with the goal of gaining a better understanding of our community’s needs and the current barriers that exist to clinical trial participation. Our future direction is to apply the lessons learned to effectively address and overcome these barriers for the proposed clinical trial, as well as future trials. Although McCarron and colleagues describe the use of a community advisory board (CAB) in developing interventions for dementia care, community-engaged strategies have not been widely implemented by multidisciplinary dementia care centers [20]. Our CE studios are unique in that they were conducted by a CEAD, and their success was largely due to the resources available to us, including our community partnership with JASA. JASA served as an invaluable collaborator by promoting and facilitating access to key stakeholders as well as a virtual platform to conduct the studios.

While introducing technology often presents challenges in older adults, JASA had implemented virtual NORC programming prior to our CE studios. They had addressed some of the common barriers to digital engagement in an older population by providing clients with the devices, IT training, and troubleshooting to facilitate participation in community activities. By the time we conducted our CE studios, JASA NORC members had a familiarity and comfort with the virtual platform that allowed us to avoid many of the challenges that would typically present themselves in introducing technology in a population of older adults. When conducting CE studios in novel populations, it would be important to assess stakeholder familiarity and access to technology before implementing a virtual CE studio and to provide support as necessary.

Even in a cohort of stakeholders who are comfortable with each other and technology, the virtual group platform still raises unique challenges to group dynamics. For example, at times, it was difficult for conversation to flow naturally because it is difficult to interpret body language virtually. Stakeholders often interrupted one another at the start of a conversation because they could not tell when someone else was about to speak. Therefore, in the virtual setting, the facilitator played an even more crucial role in promoting the flow of discussion.

Our CE studios were impacted by the fact that they occurred virtually as opposed to faceto-face. Most importantly, the length of studio was modified so that it lasted 1 h, as opposed to the 2–3 h that is described in the literature, to prevent participant fatigue [21]. CE studios typically begin with an “ice breaker” activity in order to introduce participants to one another as well as to create a comfortable environment for stakeholders to share. Due to the fact that our studios were conducted in a group already familiar with each other, we were able to omit this activity and begin with the 7-min introductory presentation. We also received minimal questions following the introductory presentation because a JASA social worker had already introduced stakeholders to the basic structure and purpose of the studio. The remaining 53 min of the 1-h session were spent discussing the 11 questions posed to the group. Eleven questions may seem difficult to discuss in the allotted amount of time, but we were successfully able to elicit feedback on each of the questions. When designing questions and considering timing, it is important to consider that some questions may require a more in-depth discussion than others. For example, the question we posed about compensation took approximately 3 min to discuss because one stakeholder proposed an answer that the other stakeholders agreed with, whereas the question we posed about how talk therapy should be described was a longer conversation with differing opinions. We greatly benefited from conducting our studios with a group familiarized with each other, the studio process and technology. It is important to take these factors into consideration when planning the length of the studio.

The feedback that we received from our stakeholders about transportation, language, and recruitment methods are known barriers to clinical trial participation. Nevertheless, when planning a clinical trial, many of these details often get overlooked or forgotten. For example, when PATH was originally studied by Kiosses and colleagues in 2016, transportation was not provided [11]. When we designed the multicenter RCT including Montefiore, transportation was again not included. We overlooked the fact that, on average, participants from Cornell in Westchester are of a higher socioeconomic status than participants from Montefiore in the Bronx, and as such, are more likely to have the ability to drive and have access to convenient parking. US Census data from 2015–2019 reports show that the median household income in Westchester County is $96,610, whereas in the Bronx the median household income is $40,088 (jasa.org). Our stakeholders reminded us that transportation was crucial to Bronx community members, and their participation was contingent on it being provided. While many variables are known to affect clinical trial participation, CE studios are helpful in determining just how important each variable is to the specific population of interest. For example, our stakeholders are older, community-dwelling adults, many of whom spend a significant amount of time in their local senior center. As such, it was important to them that we recruit in senior centers. Additionally, our stakeholders come from diverse backgrounds, many of which stigmatize therapy and psychiatric illness. As such, it was important to our stakeholders that we avoid using this terminology in favor of a less stigmatized word. Not every variable will be able to be considered when budgeting or planning for a trial, but CE studios allow researchers to take these factors into account early in trial design.

### Limitations

There were several limitations to our methodology. Though the CE studios were open to all JASA NORC members, our participants were all female. In research about older adults, women are more strongly represented due to a consistent differences in life expectancy (cdc.gov). It would have been more informative to have more ethnically and linguistically diverse participants (i.e. NH White, Hispanic, and Asian) in the CE studios. The demographic makeup of the Bronx and specifically, the location of the NORC is predominately NH Black, which limited the diversity in our stakeholders. Nonetheless, conducting the CE studios in a cohort of NH Black women did provide insight from a key population that is traditionally underrepresented in clinical research.

### Future Directions

Our CE studios were conducted in the early planning stages for a clinical trial. Stakeholder feedback regarding language, transportation, recruitment, and time commitment will be taken into consideration as we move forward in the planning stages.

Adapting the PATH trial design and intervention to be more inclusive will be an iterative process that will require a pragmatic trial design. We foresee a 6 to 12 months pilot period in which we can assess our strategies using mixed methods including CE studios, debrief interviews, and quantitative questionnaires.

Some of the immediate study design changes that we can make include decreasing the number of sessions, providing transportation, and recruiting more ethnically/linguistically concordant clinical staff. We aim to use less stigmatizing language in our recruitment materials including replacing “talk therapy” with “helpful conversations.” One of the aims of this trial will also be to develop an inclusivity toolkit which can be applied to AD and related dementia clinic trials.

While we did not have the opportunity to conduct a CE studio in more diverse cohort, the literature and our current findings suggests that the overall feedback would be similar [15, 22, 23]. Nevertheless, we plan on conducting one to two CE studios in a Spanish-speaking cohort by leveraging our relationship with another community organization that services Hispanic older adults. Given the rich feedback and existing infrastructure at JASA, we would like to institute an ongoing CAB that can provide continuous feedback throughout the rest of the planning process. Additionally, disseminating this model of virtual CE studios to other New York State CEADs may help achieve the larger goal of improving AD and related dementia clinical trial access across a wide variety of patient populations.

## Conclusion

Our CEAD successfully conducted virtual CE studios during the COVID-19 pandemic, by partnering with a community-based organization, to engage community stakeholders about clinical trial design. Centers of Excellence exist at an intersection between direct patient care and clinical research and as such, they are in a unique position to implement CE studios to better support patient access to clinical trials. CE studios can be widely implemented across a variety of specialized clinical populations.
